# Knowledge, Perceived Risk, and Preventive Behaviors amidst Covid-19 Pandemic among Dental Students in Malaysia

**DOI:** 10.3390/dj9120151

**Published:** 2021-12-14

**Authors:** Azlini Ismail, Nur Hanisah Ismail, Nur Yasrin Maisarah Abu Kassim, Widya Lestari, Ahmad Faisal Ismail, Cortino Sukotjo

**Affiliations:** 1 Department of Fundamental Dental and Medical Sciences, Kulliyyah of Dentistry, International Islamic University Malaysia, Pahang 25200, Malaysia; dr_azlini@iium.edu.my (A.I.); drwidya@iium.edu.my (W.L.); 2 Kulliyyah of Dentistry, International Islamic University Malaysia, Pahang 25200, Malaysia; nhanismail25@gmail.com (N.H.I.); yasrinmaisarah26@gmail.com (N.Y.M.A.K.); 3 Department of Pediatric Dentistry and Dental Public Health, Kulliyyah of Dentistry, International Islamic University Malaysia, Pahang 25200, Malaysia; drfaisal@iium.edu.my; 4 Department of Restorative Dentistry, College of Dentistry, University of Illinois at Chicago, Chicago, IL 60612, USA

**Keywords:** COVID-19, dental education, dental student, knowledge, perceived risk, preventive behavior, Malaysia

## Abstract

Purpose: Coronavirus disease 19 (COVID-19) has affected dental education in Malaysia. However, studies on dental students’ knowledge, perception, and behaviors with regards to COVID-19 are very limited. Thus, this study aims to determine the knowledge status, perceived risk, and preventive behaviors of dental students in Malaysia regarding COVID-19. Methods: A cross-sectional study was conducted among students from 13 dental schools across Malaysia using online questionnaires. Results: From 355 respondents, 93.5% obtained a high score of knowledge of COVID-19. Female respondents scored higher than males in perceived risks and preventive behaviors. Chinese respondents scored highest in knowledge, while Malay respondents had the highest perceived risk score. The mean preventive behavior score did not vary across ethnicity. On-campus students scored higher in knowledge and perceived risk whereas off-campus students practiced more preventive behaviors. Clinical students’ knowledge score was higher than preclinical students. Final year students scored higher in knowledge and perceived risk compared to their juniors. Conclusion: The majority of dental students have good knowledge and a high perceived risk of COVID-19, and they practiced most of the preventive behaviors. However, the latest information on this disease should be incorporated into dental schools’ curriculums and updated periodically.

## 1. Introduction

Coronavirus disease 19 (COVID-19) was initially identified during the pneumonia outbreak in Wuhan City, Hubei Province, China [1]. After the local spread, it became a global outbreak and was declared a pandemic on 11th March 2020 by the World Health Organization [2]. The first index case of COVID-19 in Malaysia was announced on 25th January 2021 [3]. On 18th March 2020, the Malaysia government had to employ a Movement Control Order as a restriction intervention to curb the uncontrolled situation. After the drastic measure, the COVID-19 situation in Malaysia was getting better, with the lowest record of only one import case on 1 July 2020. However, by 20 September 2020, Malaysia was in the midst of the third wave of infections, in which cases were spurting non-stop. As of 12 September 2021, 224.11 million cases were confirmed globally, with 4.6 million deaths, and in Malaysia, there were 1,975,074 confirmed cases, with more than 20,711 fatalities found across all states [4]. 

During this pandemic, not only was the public’s health in danger, but Malaysia’s economic sector, education system, and healthcare services, including dental institutions were majorly impacted [5]. Dental professionals are among the forefronts at risk because their working environment can potentially create a virus-laden aerosolized environment [6]. Plaque or calculus are the major sources of bacteria and virus in the oral fluids. These pathogens can be aerosolized during dental procedures that lead to airborne contamination thus transmitting airborne diseases including SARS-CoV-2 which has been detected in the saliva of infected individuals [7,8]. This has raised a high level of health risk perception among dental practitioners, including clinical staff, dental educators, and students regarding dental practice during the COVID-19 outbreak [9]. Because of this matter, some extra precautionary measures such as the usage of a higher level of personal protective equipment (PPE) has increased drastically during dental practice [9]. Besides that, the patient’s screening and clinic’s infrastructure have been improvised from time to time, according to the latest government’s standard operating procedure (SOP); all of these are integral to reduce the infection risk posed by this virus. In a way, COVID-19 has brought improvements in dental practice especially in terms of infection prevention and reactions to future contagious diseases by means of the reinforcement of PPE implementation and development of more effective infection control measures [10].

According to the Centre of Disease Control and Prevention (CDC), dental school educational activities are bound to low-risk activities such as online case study as well as higher risk activities such as aerosol-generating procedures in the clinic [6]. By means of this, dental students are also exposed to the same threats as dental practitioners since they are treating patients during their clinical years to complete their clinical requirements. There are currently 13 institutions in Malaysia, either government or private higher institutions, that offer dentistry as a formal degree. Given the context that the dental students have a high risk of being in close contact with affected people during the clinical dental practice amidst this pandemic, they need to have sufficient knowledge on COVID-19 as this is believed to influence their precautionary behaviors. Unfortunately, a study regarding Malaysian’s dental student knowledge about COVID-19 was limited to a particular university in this region [3]. Therefore, this study aims to extend the research in determining the knowledge status of dental students in Malaysia regarding COVID-19′s mode of transmission, diagnosis, treatment, signs, and symptoms; their perceived risk of COVID-19 as threats to them as an individual or towards their family; and their preventive or precautionary behaviors to break the COVID-19 chain of infection during this pandemic.

## 2. Materials and Methods

### 2.1. Ethical Aspect

Ethical approval was obtained from the IIUM Research Ethics Committee on 21st January 2021 (Ref. No: IREC 2021-013). 

### 2.2. Study Design

This cross-sectional study was conducted among dental students in Malaysia through an online survey by creating a Google Form to allow respondents to access the questionnaire with ease. 

### 2.3. Sampling Method

The sampling method used was convenience sampling which is a type of non-probability sampling. By using this sampling method, the members of the population freely participated based on their availability and accessibility to the survey instrument.

### 2.4. Inclusion and Exclusion Criteria

The inclusion criteria for this study were dental students in Malaysia. The exclusion criteria for this study were respondents who submitted incomplete survey questionnaires or denied the consent to participate.

### 2.5. Sample Size

The target population was dental students from public and private universities in Malaysia. From a calculation using Raosoft^®^ software (Raosoft Inc, Seattle, WA, USA) (http://www.raosoft.com/samplesize.html) (accessed on 1 December 2020), the recommended sample size for this study was 347 students based on the population size of 3514 Malaysian dental students with a confidence level of 95 and a 5% acceptable margin of error. 

### 2.6. Data Collection

The study was conducted through an online survey using Google Form. Beginning on 11 March 2021, this study was completed on 30 April 2021. The questionnaire consisted of four sections as follows:

- Section 1: Demographic background

This section consisted of eight items, including age, gender, nationality, ethnicity, current location, year, and phase of study (clinical or preclinical).

- Section 2: Knowledge on COVID-19

This section was adapted based on the questionnaire from a local study by Wee et al. (2020) and a multi-country study by Ammar et al. (2020) with some modifications according to the Ministry of Health Malaysia guidelines [3,11]. There were six items that consisted of ‘True’ and ‘False’ questions. These items evaluated the knowledge of COVID-19 related to the incubation period, vulnerable groups, signs and symptoms, transmission routes, preventive measures, diagnosis, and treatment with a total score of 26 points. Correct responses to questions were summed up and a final percentage was calculated on a 100-point scale. A score >80% was designated as high, 60–80% as moderate, and <60% as poor knowledge [3].

- Section 3: Perceived risk

This section used the COVID-19 Perceived Risk Scale or CPRS, a scale developed for assessing COVID-19-related perceived risk [12]. The CPRS consisted of eight items, each rated on a five-point Likert scale ranging between 1 (negligible) and 5 (very large). The section included a cognitive dimension and emotional dimension. Higher scores reflected higher levels of personal perceived risk related to COVID-19.

- Section 4: Preventive behaviors

This section was adapted based on the questionnaire from a local study by Wee et al. (2020) [3]. There were 17 statements with a five-point Likert scale ranging between 1 (certainly no) and 5 (definitely yes). All items focused on the preventive practices of respondents during the COVID-19 pandemic. A high score reflected a good level of preventive behaviors during the pandemic.

### 2.7. Testing Validity and Reliability of Questionnaire

A face-to-face validity was conducted by asking five dental students at the International Islamic University Malaysia (IIUM) to answer the questionnaire. This phase was conducted to test and judge the items of the study instrument in terms of clarity of wording, readability, and likelihood of respondents answering the questions. The questionnaire was edited accordingly after the face-to-face validation. A pilot study was then carried out among 42 IIUM dental students as a preliminary analysis to evaluate the reliability of the questionnaire using Cronbach’s alpha.

### 2.8. Statistical Analysis

Data were analyzed using Microsoft Excel and SPSS statistical software (Version 25.0). Descriptive characteristics including age, gender, nationality, ethnicity, current location, institution, and year and phase of the study (clinical or preclinical) were analyzed. Differences between mean scores of knowledge, perceived risk, and preventive behavior scores between different sociodemographic characteristics of dental students were tested using the Mann–Whitney U test for two groups comparison and Kruskal Wallis test for three or more groups comparison since the data were not normally distributed. The level of significance was set at 0.05 with 95% confidence intervals.

## 3. Results

### 3.1. Demographic Data

This study comprised a total of 355 dental students in Malaysia. The sociodemographic characteristics of the respondents are presented in Table 1. The majority of respondents were female (76.9%), Malaysian students, and Malaysian ethnicity. The mean age was 22.40 years. Most of them (64.8%) stayed on campus. The majority of respondents were year 4 (31.0%), followed by year 5 (21.1%). Clinical students accounted for the highest proportion (62.8%) of the total respondents.

### 3.2. Knowledge of COVID-19

Table 2 shows all true–false statements which were given to test the knowledge of dental students of COVID-19. The majority of respondents answered correctly for all questions asked except for a few questions regarding COVID-19 transmission (76.3% selected ‘True’ for touching body fluid of an infected patient, 78.0% selected ‘False’ for touching domestic pets, and 74.9% selected ‘True’ for touching objects that an infected person has touched) and possible signs or symptoms of COVID-19 infection (67.9% selected ‘True’ for muscle or joint pain, 61.7% selected ‘False’ for dry mouth).

### 3.3. Perceived Risk 

Table 3 comprises five questions on the perceived risks of COVID-19. Most respondents were worried and extremely worried about the possibility of their family members contracting COVID-19 and the disease becoming a health issue. In total, 33.5% were worried and extremely worried (33.0%) about themselves contracting COVID-19. The majority of the respondents perceived moderate (36.9%) to high (29.0%) chances of acquiring COVID-19 compared to other persons. Most of the respondents perceived a low (36.3%) to moderate (37.5%) risk of dying from COVID-19.

### 3.4. Preventive Behaviors

Table 4 depicts the preventive behaviors practiced by the respondents during the COVID-19 pandemic. The majority of the students practiced preventive behaviors against COVID-19. Besides that, 37.2% would certainly wash their hands regularly according to the recommended time; however, 29.9% were indecisive about practicing cleaning and disinfecting items that can be easily touched by hands. In total, 53.5% of the respondents would not usually greet people with a handshake, while 67.3% would not usually greet people with a hug.

### 3.5. Knowledge, Perceived Risk, and Preventive Behavior Scores

Table 5 summarizes the average percentages of knowledge and scores for perceived risk and preventive behaviors. In total, 93.5% of respondents scored a high score for knowledge of COVID-19, 6.50% obtained an average score, while none of the respondents scored poorly in this section. Mean scores for students’ knowledge about COVID-19 was 23.29 ± 1.76, out of 25 total scores. The highest score attained was 100%, and the lowest attained score was 65.4%. Concerning perceived risk and preventive behaviors, overall respondents scored a mean of 18.35 ± 3.05 out of an overall score of 25, and 57.03 ± 5.67, out of an overall score of 65, respectively. 

### 3.6. Association between Demographic Background and Each Domain Score

Table 6 shows the mean score of knowledge, perceived risk, and preventive behaviors, according to the sociodemographic characteristics of dental students. The mean scores of perceived risks were significantly higher among female respondents (18.66 ± 2.914) than males (17.33 ± 3.293) (*p* = 0.001). Similarly, females had a significantly higher mean preventive behavior score (57.70 ± 5.318) than the males (54.80 ± 6.235) (*p* = 0.000). All dependent variables were not significantly different (*p* > 0.05) with nationality (Malaysian or international). With regards to ethnicity, significant differences were found with the knowledge and perceived risk scores among the respondents from various ethnic groups. Chinese respondents had the highest mean knowledge score among ethnic groups with 23.87 ± 1.556 as compared to other groups (*p* = 0.0190). In contrast to knowledge, Malay respondents (18.74 ± 2.905) had a better perceived risk score compared to other groups (*p* = 0.000). In terms of preventive behaviors, the mean score was not significantly different (*p* > 0.05) across ethnicities. Interestingly, students who were on campus scored significantly higher in knowledge (*p* = 0.013), and perceived risk score (*p* = 0.010), but significantly lower for preventive behaviors (*p* = 0.009). Knowledge and perceived risk score significantly differed according to the year and phase of study. The mean score for knowledge was higher among clinical students as compared to preclinical students (*p* = 0.000). Year 5 students had the highest knowledge mean score (*p* = 0.000) and the best perceived risk scores (*p* = 0.041), followed by year 4, year 3, year 2, and year 1 students. No significant difference was found in the mean preventive behavior scores when comparisons were made between students across years and phases of study.

## 4. Discussion

Dental education is facing a great impact due to the sudden halt in the educational activities because of the COVID-19 pandemic. The majority of dental schools worldwide suspended activities to minimize the transmission of the virus [13]. New oral health policies were implemented including strict infection control measures, postponing non-emergency treatment, and upgrading ventilation systems [14]. Besides that, the clinical competence and academic performance of dental students were negatively affected due to the suspension of the usual face-to-face teaching activities [9]. As COVID-19 stays, and with the emergence of a vaccination plan in Malaysia, dental schools have re-opened following the need of students to complete their clinical requirements for graduation. However, at this point of time, the knowledge status, perceived risk, and preventive behaviors amongst dental students in Malaysia are not yet known. 

To the best of our knowledge, this is the first study in Malaysia that discusses knowledge, perceived risk, and preventive behaviors during the COVID-9 pandemic among dental students throughout Malaysia. Our study provides an overview of the knowledge of dental students who have a high risk of infection during the clinical dental practice, which involves rotative instruments and aerosol build-up. Lacking in knowledge, perceived risk, and preventive measures can threaten dental staff and the patients’ safety and health which may deteriorate the health care system.

The current study showed that most respondents (93.5%) had a high level of knowledge by scoring 80% and above, while the rest showed a moderate level of knowledge (6.5%). Similarly, the high knowledge score was actually found in other studies among dental students from Palestine, Saudi Arabia, and Austria [15,16,17]. In another study conducted in Jordan, knowledge scores were significantly higher for medical and dental students than other disciplines such as pharmacy and nursing [18]. However, in comparison with a previous local study in Malaysia, it was demonstrated that only 55.2% of medical and dental students had high knowledge with scores of over 80%, while the rest had moderate knowledge about COVID-19 [3]. The knowledge score was lower in this study compared to our current study, perhaps because this prior study was conducted in an earlier period of the COVID-19 pandemic in Malaysia. At that time, the knowledge of COVID-19 was by far still lacking and not well-disseminated.

In our study, dental students had good knowledge of COVID-19 across multiple topics. The correct responses in all questions were 70% and above, showing that all students possessed a good understanding of COVID-19. However, regarding the symptoms related to COVID-19, there were two symptoms that less than 70% of students correctly identified: dry mouth (61.7%) and muscle or joint pain (67.9%). This is congruent with a previous study by Alawia et al. (2021) in which the questions about muscle or joint pain were correctly answered only by 50.2% of the students [15]. In fact, the WHO and CDC reported muscle or joint paint as the less-common symptoms of COVID-19. Even though dry mouth was not a symptom directly associated with COVID-19 infection, Freni et al. (2020) reported the signs of dry mouth in 32% of the COVID-19 patients, and in most cases, it occurred before other symptoms of the disease [19]. Most respondents recognized that antibiotics are ineffective in preventing COVID-19, probably because they understood that COVID-19 is a viral disease [15,20].

Regarding sociodemographic characteristics and knowledge, our findings show no significant difference between male and female respondents’ knowledge of COVID-19. Both groups recorded almost similar knowledge mean scores. This is aligned with the study conducted in a local university in Malaysia that found no significant association between gender and knowledge of COVID-19 [3]. On the contrary, the results of the study among dental students in Saudi Arabia showed a statistically significant difference in males’ and females’ knowledge, with females scoring better than males [16]. Another study in India also revealed significant differences in the mean scores of knowledge, where male dental students had higher mean knowledge scores than female students [21]. 

Overall, the knowledge score for the students significantly differed based on ethnicity, current location, year of study, and phase of study. In the present study, there was a significant difference between preclinical students and clinical students in the knowledge score, with the mean score being lower among preclinical students. Our results are in line with other research carried out among dental students in Turkey. There, they concluded knowledge, attitudes, and practices regarding COVID-19 did present a significant difference between preclinical and clinical students [22]. This is possibly due to the fact that clinical students who had started treating patients had better knowledge and attitudes towards infectious diseases owing to the extent of time they spent at the clinical setting, where they worked at a higher risk of exposure.

Perception towards risk, severity, and susceptibility plays an important role in determining an individual’s preventive behaviors. [23]. In our study, the perceived risk towards COVID-19 was assessed by analyzing respondents’ perceptions and comprehension on the risk imposed by COVID-19 on one’s health in which about two-thirds of the respondents considered COVID-19 as a deadly disease. This is unlike a population-based survey in Iran, where only half of their respondents perceived COVID-19 as a serious issue, and this was believed to be associated with the lower level of knowledge during the conduct of that study [24]. We found that a higher proportion of respondents were concerned about their family’s health, while less than a quarter assumed themselves at risk of contracting COVID-19. Consistent with a study from China, college students were more worried of their elder family members than themselves because the elderly are more vulnerable and have worse prognoses with COVID-19 infections [25]. We also found that almost half of the respondents assumed that they had the possibility of dying due to COVID-19. Similarly, 92.6% (n = 3720) of people in Anhui province, China, were agitated with the spread of COVID-19 [26]. These observations were supported by a few laboratory findings that proved an increase in death anxiety and preventive behaviors as a reflection of the current epidemics or virus outbreaks [27]. Death tolls of COVID-19 on the daily news were also considered as a factor that built fears of death among the public [28]. The increase in dental students’ perceived risks were due to the novel nature of the virus, the lack of vaccines or effective treatments at the early pandemic phase, being near to untested patients, the high risk of inhaling respiratory droplets and saliva in the confined space of the dental unit, and the lack of awareness regarding the disease and precautionary measures among patients [9,29].

Our study also revealed that more female students showed higher scores in perceived risks and preventive behaviors, with average scores of 18.66 and 57.70, respectively, as compared to male students with average scores of 17.33 and 54.80, respectively. In corroboration with this finding, a survey among medical and dental students of a local university in Malaysia showed a relatively higher number of female students who practiced positive attitudes and preventive behaviors than male students [3]. Higher risk perceptions among females can be described by their unfeasible thoughts, gender socialization, and high sensitivity towards the health risks [25,30]. Few previous reports showed unparallel findings on how students’ locations were affecting their perceived risks. A former study explained that the students from the outbreak region had higher risk perception than the non-epidemic region, while our findings revealed that students who stayed on the campus had a higher perceived risk mean score than those at home [25]. Besides that, our survey found a significant difference in the risk perception of students in the clinical phase compared with students in the preclinical phase, in which the former had a higher mean score of perceived risks than the latter. Students with comprehensive knowledge were more aware of the risk and had a higher risk perception of COVID-19 [25]. Hence, a good understanding of this pandemic directly affects the perception of risk and preventive practices.

In accordance with preventive measures recommended by the WHO, generally students performed good preventive behaviors. The majority of the respondents would definitely wear a mask and practice physical distancing in public, and they would follow health authorities’ instructions. In order to break the chain of infection, about half of the respondents practiced regular hand washing and disinfecting items that can be easily touched with hands. Generally, the respondents strongly agreed that handshaking and hugging should not be practiced during this death-dealing situation. A study conducted on preventive health behaviors during the COVID-19 pandemic based on the Health Belief Model among Egyptians showed that highly-educated persons have better preventive behavior. This is because they have higher knowledge about the disease, better-perceived susceptibility, and low perceived barriers [31]. The results also proved that students were eager to keep themselves updated with COVID-19 updates either locally or globally. 

Our study also highlighted that COVID-19 protective measures significantly differed by gender and students’ current location. Few previous studies proved that female students practiced more preventive behaviors compared to male students [3,32]. However, a study based in India had a different view and concluded that women in Bihar, India had less knowledge; therefore, they were less likely to practice preventive behaviors due to limited and challenging access to receive accurate information. Regarding students’ current location, students living off-campus had higher preventive behavior scores than students living on-campus. Those staying on campus perhaps generally regard the campus as a safe environment, and those staying off campus generally need to travel, which exposes them to a higher risk of infection. No significant difference was found between preventive behavior scores of those clinical and preclinical students. In contrast, a study among medical students in Jordan reported that clinical students practiced more protective measures, such as using disinfectants, than the preclinical students [33].

Overall, this study has some limitations in that the convenience sampling method used in this study may reduce the statistical representativeness. However, convenience sampling is the most feasible method for this study to sample dental students across the whole country as there is no centralized listing of all dental students available. Despite this, this study managed to acquire dental students from most dental schools in Malaysia through dental students societies representatives. Secondly, the cross-sectional design of this study may not always cover the knowledge on COVID-19 during the pandemic. Another challenge is that the knowledge on this area is continually evolving as more new findings are discovered each day. Even though this study showed that dental students in Malaysia possessed good knowledge on COVID-19, with the continued growth of knowledge on COVID-19, dental students should keep abreast of all the recent developments. 

## 5. Conclusions

In summary, most dental students in Malaysia have good knowledge about COVID-19. Hence, their perceived risk of the disease is high, and they are effectively practicing preventive behaviors. However, with the accumulating new knowledge on COVID-19, the latest information on this disease should be incorporated into dental schools’ curriculums and updated from time to time.

## Figures and Tables

**Table 1:** Sociodemographic background.

Variables	Frequency	
	N (Total = 355 Respondents)	%
**Gender**		
Male	82	23.1
Female	273	76.9
**Age**		
<22	121	34.0
22–25	229	64.6
>25	5	1.4
**Nationality**		
Malaysian	351	98.9
International	4	1.1
**Ethnicity**		
Malay	270	76.1
Chinese	60	16.9
Indian	14	3.9
Others	11	3.1
**Current location**		
Off campus	125	35.2
On campus	230	64.8
**Year of study**		
1	72	20.3
2	53	14.9
3	45	12.7
4	110	31.0
5	75	21.1
**Phase of study**		
Preclinical	132	37.2
Clinical	223	62.8

**Table 2:** Knowledge of COVID-19.

Statements	Correct Responses, N (%)
Incubation period of COVID-19 is up to 14 days. *	326 (91.8)
**Vulnerability to COVID-19:**	
Only older adults can become infected with COVID-19.	331 (93.2)
Older adults are the group with highest mortality when infected with COVID-19. *	322 (90.7)
Those with other health problems more likely to die from COVID-19 than those without any other health problems. *	344 (96.9)
**Regarding COVID-19 diagnosis and treatment:**	
It can be diagnosed by PCR test on samples collected from nasal/pharyngeal swab. *	353 (99.4)
The disease can be solely treated by using antiviral drugs.	299 (84.2)
**COVID-19 can be transmitted through:**	
Direct contact with aerosol splattered during a dental procedure in an infected patient. *	337 (94.9)
Inhaling air from an infected patient. *	285 (80.3)
Touching body fluids of an infected patient. *	271 (76.3)
Touching domestic pets (e.g., cat, dog, etc.).	277 (78.0)
Touching objects that have been touched by an infected person. *	266 (74.9)
Breathing in droplets exhaled or coughed from an infected person. *	355 (100.0)
**Possible signs or symptoms of COVID-19 infection:**	
Nose bleeds	338 (95.2)
Cough *	353 (99.4)
Dry mouth	219 (61.7)
Fever *	351 (98.9)
Muscle or joint pain *	241 (67.9)
Shortness of breath *	348 (98.0)
Sore throat *	341 (96.1)
Loss of smell and taste sensation *	344 (96.9)
**Ways to prevent COVID-19 infection:**	
Getting a vaccination against pneumonia	250 (70.4)
Wearing a facemask *	354 (99.7)
Washing your hands *	355 (100.0)
Avoiding close contact with people who are sick *	351 (98.9)
Taking antibiotics	305 (85.9)
Avoiding touching your eyes, nose and mouth with unwashed hands *	352 (99.2)

* Correct statement.

**Table 3:** Perceived risk of COVID-19.

Questions	1 N (%)	2 N (%)	3 N (%)	4 N (%)	5 N (%)	Mean	SD
How worried are you about your family member contracting COVID-19?	2 (0.6)	10 (2.8)	26 (7.3)	90 (25.4)	227 (63.9)	4.49	0.797
How worried are you about COVID-19 emerging as a health issue?	4 (1.1)	5 (1.4)	36 (10.1)	129 (36.3)	181 (51.0)	4.35	0.807
How worried are you about contracting COVID-19?	8 (2.3)	26 (7.3)	85 (23.9)	119 (33.5)	117 (33.0)	3.88	1.026
Compared to other persons, what would you say about your chances for acquiring COVID-19?	14 (3.9)	68 (19.2)	131 (36.9)	103 (29.0)	39 (11.0)	3.24	1.012
What is the likelihood that you would die from COVID-19?	63 (17.7)	129 (36.3)	133 (37.5)	20 (5.6)	10 (2.8)	2.39	0.937

1 (negligible), 5 (very large), SD: standard deviation.

**Table 4:** Preventive behaviors against COVID-19.

Statement	1 N (%)	2 N (%)	3 N (%)	4 N (%)	5 N (%)	Mean	SD
I usually put on a face mask whenever I go out in public.	0 (0)	0 (0)	2 (0.6)	16 (4.5)	337 (94.9)	4.94	0.807
If I find that I was in contact with an infected person, I agree to be quarantined as instructed by health authorities.	1 (0.3)	1 (0.3)	9 (2.5)	43 (12.1)	301 (84.8)	4.81	0.507
If I find that I was in contact with an infected person, I will inform the health authorities.	1 (0.3)	3 (0.8)	18 (5.1)	55 (15.5)	278 (78.3)	4.71	0.628
If I have any of the symptoms associated with the disease, I will inform the health authorities.	0 (0)	4 (1.1)	24 (6.8)	68 (19.2)	259 (73.0)	4.64	0.659
I practice physical distancing whenever I go out in public.	0 (0)	2 (0.6)	25 (7.0)	123 (34.6)	205 (57.7)	4.50	0.653
I am willing to undergo a COVID-19 test although I am not instructed to (e.g., back from travelling or red zone).	6 (1.7)	10 (2.8)	41 (11.5)	74 (20.8)	224 (63.1)	4.41	0.920
I discuss COVID-19 prevention with my family and friends.	0 (0)	11 (3.1)	35 (9.9)	117 (33.0)	192 (54.1)	4.38	0.788
I follow the updates about the spread of the virus in Malaysia.	3 (0.8)	17 (4.8)	38 (10.7)	126 (35.5)	171 (48.2)	4.25	0.891
I wash my hands regularly and for enough period of time.	3 (0.8)	16 (4.5)	77 (21.7)	127 (35.8)	132 (37.2)	4.04	1.026
I follow the updates about the spread of the virus worldwide.	7 (2.0)	31 (8.7)	100 (28.2)	91 (25.6)	126 (35.5)	3.84	1.068
I practice cleaning and disinfecting items that can be easily touched with hands (i.e., door handles, lift buttons, and table surfaces).	7 (2.0)	44 (12.4)	106 (29.9)	103 (29.0)	95 (26.8)	3.66	0.797
When I meet other people, I usually greet them with a handshake. *	190 (53.5)	112 (31.5)	31 (8.7)	15 (4.2)	7 (2.0)	1.70	1.012
When I meet other people, I usually greet them with a hug. *	239 (67.3)	85 (23.9)	20 (5.6)	9 (2.5)	5 (0.6)	1.45	0.937

1 (certainly no), 5 (definitely yes), * Statements indicating behaviors that should be avoided. SD: standard deviation.

**Table 5:** Knowledge of COVID-19, perceived risk, and preventive behaviors of dental students in Malaysia.

Variable	N (%)
**Knowledge Scores (0–100%)**	
High (>80%) (21–26)	332 (93.5)
Moderate (60–80%) (17–20)	23 (6.50)
Poor (<60%) (0–16)	0 (0.0)
Minimum	17 (65.4)
Maximum	26 (100)
Mean ± SD	23.29 ± 1.76
**Perceived Risk Scores (5–25)**	
Mean ± SD	18.35 ± 3.05
**Preventive Behaviors Scores (13–65)**	
Mean ± SD	57.03 ± 5.67

**Table 6:** Knowledge, perceived risk, and preventive behaviour scores according to sociodemographic profiles of dental students.

Sociodemographic Characteristics	N	Knowledge Score		Perceived Risks Score		Preventive Behaviors Score	
		Mean (SD)	*p*-Value	Mean (SD)	*p*-Value	Mean (SD)	*p*-Value
**Gender**			0.517		0.001 *		0.000 *
Male	82	23.22 (1.722)		17.33 (3.293)		54.80 (6.235)	
Female	273	23.31 (1.768)		18.66 (2.914)		57.70 (5.318)	
**Nationality**			0.911		0.263		0.731
Malaysian	351	23.29 (1.761)		18.36 (3.051)		57.03 (5.672)	
International	4	23.50 (1.291)		17.00 (3.367)		56.50 (5.972)	
**Ethnicity**			0.019 *		0.000 *		0.569
Malay	270	23.13 (1.810)		18.74 (2.905)		56.91 (5.411)	
Chinese	60	23.87 (1.556)		16.68 (3.392)		57.55 (6.080)	
Indian	14	23.43 (1.342)		18.71 (2.164)		57.07 (8.880)	
Others	11	23.82 (1.168)		17.27 (2.573)		57.09 (5.147)	
**Current location**			0.013 *		0.010 *		0.009 *
On campus	125	23.47 (1.686)		18.69 (2.905)		56.39 (6.013)	
Off campus	230	22.96 (1.838)		17.72 (3.224)		58.21 (4.768)	
**Year of study**			0.000 *		0.041 *		0.565
Year 1	72	22.18 (1.841)		17.79 (3.131)		58.13 (4.850)	
Year 2	53	22.58 (1.737)		17.72 (3.416)		56.81 (6.382)	
Year 3	45	23.60 (1.468)		18.04 (2.611)		56.73 (4.721)	
Year 4	110	23.74 (1.572)		18.71 (3.081)		56.66 (6.055)	
Year 5	75	24.01 (1.419)		18.99 (2.773)		56.84 (5.803)	
**Phase of study**			0.000 *		0.004 *		0.093
Preclinical	132	22.40 (1.786)		17.68 (3.298)		57.64 (5.504)	
Clinical	223	23.82 (1.512)		18.74 (2.832)		56.66 (5.742)	

* *p* < 0.05, Mann–Whitney U test for two groups comparison and Kruskal Wallis test for three or more groups comparison, SD: standard deviation.

## Data Availability

Data of the study will be shared upon request to the corresponding author.
